# Taurisolo®, a Grape Pomace Polyphenol Nutraceutical Reducing the Levels of Serum Biomarkers Associated With Atherosclerosis

**DOI:** 10.3389/fcvm.2021.697272

**Published:** 2021-07-19

**Authors:** Giuseppe Annunziata, Roberto Ciampaglia, Maria Maisto, Maria D'Avino, Domenico Caruso, Gian Carlo Tenore, Ettore Novellino

**Affiliations:** ^1^NutraPharmaLabs, Department of Pharmacy, University of Naples Federico II, Naples, Italy; ^2^Department of Internal Medicine, Hospital Cardarelli, Naples, Italy

**Keywords:** atherosclerosis, TMAO, oxidative stress, grape polyphenols, nutraceutical

## Abstract

Trimethylamine-*N*-oxide (TMAO) is a gut microbiota-derived metabolite recognized as strongly related to cardiovascular diseases (CVD), mainly increasing the risk of atherosclerosis development. Currently, no pharmacological approaches have been licensed for reduction of TMAO serum levels and conventional anti-atherosclerosis treatments only target the traditional risk factors, and the cardiovascular risk (CVR) still persist. This underlines the need to find novel targeted strategies for management of atherosclerosis. In this study we tested the ability of a novel nutraceutical formulation based on grape pomace polyphenols (Taurisolo®) in reducing both the serum levels of TMAO and oxidative stress-related biomarkers in humans (*n* = 213). After chronic treatment with Taurisolo® we observed significantly reduced levels of TMAO (−49.78 and −75.80%, after 4-week and 8-week treatment, respectively), oxidized LDL (oxLDL; −43.12 and −65.05%, after 4-week and 8-week treatment, respectively), and reactive oxygen species (D-ROMs; −34.37 and −49.68%, after 4-week and 8-week treatment, respectively). On the other hand, no significant changes were observed in control group. Such promising, the results observed allow indicating Taurisolo® as an effective nutraceutical strategy for prevention of atherosclerosis.

**Clinical Trial Registration:** This study is listed on the ISRCTN registry with ID ISRCTN10794277 (doi: 10.1186/ISRCTN10794277).

## Introduction

Atherosclerosis is among the main causes of mortality worldwide (1, 2). Several risk factors have been associated with this chronic artery disease, including hypertension, dyslipidemia, obesity, diabetes and cigarette smoking (1, 2). However, recently trimethylamine-*N*-oxide (TMAO) has been recognized as novel proatherogenic factor closely involved in atherosclerosis development and progression, beyond the other historically-known risk factors (2–8). This was confirmed by clinical trials (3, 9).

TMAO is a bioactive gut microbiota-derived metabolite produced from the metabolism of food components, mainly dietary choline, betaine, and carnitine (2, 3, 10). More specifically, selected bacterial strains in gut microbiota (that express target genes, including TMA lyases, carnitine oxygensae and betaine reductase) produce trimethylamine (TMA) that, in turn, is oxidized to TMAO by flavin monooxygenase 3 (FMO3) in liver (2). In this sense, clinical trials reported that alterations in microbiota composition are responsible for increased TMAO levels in response to the diet (11). The clinic interest for this metabolite derives from emerging evidence suggesting its role in pathophysiology of cardiovascular diseases (CVD) (with particular interest in atherosclerotic risk) (8, 12) and other chronic pathological conditions (13–15).

It is widely reported in literature that TMAO is closely linked to oxidative stress (OxS), as it is a highly oxidant and reactive molecule (16). With OxS refers to an unbalance between production and clearance of oxidants, mainly reactive oxygen species (ROS) (17). It has been reported that various pathological conditions, including obesity, diabetes, hypertension or dyslipidemia, are responsible for increasing levels of ROS (18, 19). Similarly, studies reported the strong relationship between OxS and CVD, including atherosclerosis (20–22). More specifically, atherosclerotic plaque formation is triggered by molecular changes induced by inflammation (i.e., increased circulating levels of cytokines) and OxS (i.e., increased circulating levels of ROS), resulting in generation of oxidized-LDL (oxLDL) that accumulate in the sub-endothelium. oxLDL, in turn, stimulate the production of adhesion molecules, with recruitment of monocytes and T-cells, resulting in release of pro-inflammatory cytokines and ROS. Main consequences of this process are apoptosis and formation of foam cells, with consequent atherosclerotic plaque production (23). It has been noted that TMAO plays a crucial role in atherosclerosis development acting on foam cell formation, endothelial dysfunction, plaque instability and platelet activation (2).

Taking into account this evidence, thus, the use of therapeutic strategies aimed to contrast the OxS is desirable, although no specific antioxidant treatment has been recommended to prevent cardiovascular complications (16). Similarly, conventional anti-atherosclerosis pharmacological treatments only target the traditional risk factors (2), and the cardiovascular risk (CVR) still persist (24, 25), underlining the need to find novel targeted strategies for management of atherosclerosis. In this sense, the use of nutraceutical products with antioxidant properties (that are generally characterized by no side-effects and no interactions with drugs) might be preferable.

Among the food-derived bioactive compounds, polyphenols, the largest class of phytochemicals (26), emerge for their cardio-protective effects, exerted *via* different mechanisms of action, mainly antioxidant and anti-inflammatory activities (27).

Aim of the study was to evaluate the effect of a novel nutraceutical formulation based on grape pomace polyphenolic extract (registered as Taurisolo®) in reducing the serum levels of TMAO, oxLDL, and reactive oxygen metabolites (ROMs) in human. The rationale behind this aim derives from previous evidence demonstrating the ability of polyphenols to reduce TMAO levels. In particular, very recently published *in vitro* experiments demonstrated the ability of gallic acid and chlorogenic acid to reduce significantly the production of TMA in fecal slurry (28). Also, Chen and colleagues reported the TMAO-reducing effect of resveratrol in animal model (29). These referred polyphenols are present in Taurisolo® composition. Moreover, it is historically known that polyphenols exert a marked antioxidant activity through direct and indirect mechanisms (30), including free radical scavenging. In particular, through their benzene ring-bound hydroxyl groups, polyphenols transfer an electron to free radicals, acting, thus, as “electron donors” (31, 32). This property of polyphenols is responsible for reduction of OxS in biological systems. Overall, this evidence strongly supported our aim to test Taurisolo®.

## Methods

### Study Population and Protocol

Study participants were recruited by public advertisement on a local newspaper published in January-April 2019. Subjects aged 18–75 years with body mass index (BMI) ≥ 18.5 kg/m^2^ were eligible for enrolment. Exclusion criteria were: underweight (BMI <18.5 kg/m^2^), cancer, hepatic disease, renal disease, heart diseases, type 1 diabetes mellitus, family history of chronic diseases, drug therapy or supplement intake containing grape polyphenols, heavy physical exercise (> 10 h/week), pregnant women, women suspected of being pregnant, women who hoped to become pregnant, breastfeeding, birch pollen allergy, use of vitamin/mineral supplements 2 weeks prior to entry into the study and donation of blood <3 months before the study.

Subjects received oral and written information concerning the study before they gave their written consent. Protocol, letter of intent of volunteers, and synoptic document about the study were submitted to the Scientific Ethics Committee of AO Rummo Hospital (Benevento, Italy). The study was approved by the committee (protocol 123512 of 18/06/2018) and carried out in accordance with the Helsinki declaration of 1964 (as revised in 2000). This study is listed on the ISRCTN registry (www.isrctn.com) with ID ISRCTN10794277 (doi: 10.1186/ISRCTN10794277). The subjects were asked to make records in an intake-checking table for the intervention study and side effects in daily reports.

This study was designed as a monocentric, double-blind, randomized, placebo-controlled, 2-arm parallel-group trial. The study duration was 14 weeks: subjects underwent 2-week run-in period (with administration of capsules containing only excipients as placebo), followed by 8 weeks of intervention (active or placebo), and 4 weeks of follow-up. The examinations were performed in an outpatient setting. Clinic visits and blood sampling were performed after 12 h of fasting at weeks 0, 2, 6, 10, and 14. Subjects were informed not to drink alcohol or perform hard physical activity 48 h prior to blood sampling. All blood samples were taken in the morning and immediately after measurement of heart rate and blood pressure. Blood samples were collected in 10-mL EDTA-coated tubes (Becton–Dickinson, Plymouth, UK) and plasma was isolated by centrifugation (20 min, 2,200 g, 4°C). All samples were stored at −80°C until analysis. In addition to these five clinic visits, six standardized telephone interviews were performed every 14 days starting from the first clinic visit, to verify compliance and increase protocol adherence. In particular, these interviews reminded patients to complete their intake-checking table for the intervention study and to record any treatment discontinuation, or adverse events they might have experienced in the meantime (which were also documented regularly on the case report forms during each telephone and clinic visit). All patients underwent a standardized physical examination, assessment of medical history (for up to 5 years before enrolment), laboratory examination, measurement of blood pressure and heart rate, and evaluation of BMI. At each clinic visit, patients had to complete three self-administered questionnaires on quality-of-life aspects, and their diaries were checked for data completeness and quality of documentation to ensure patient comprehension of the diary items.

A total of 380 patients were enrolled. If a patient dropped out before the intervention period, he or she was replaced by the next eligible patient enrolled at the same center. Subjects were randomly allocated into two intervention group: active group (400 mg Taurisolo® twice daily) or placebo (400 mg maltodextrins twice daily). Patients, clinicians, core laboratories, and trial staff (data analysts, statisticians) were blind to treatment allocation. Additional information concerning the study protocol, including study procedures and statistical analyses, are detailed in the Supplementary Materials.

### Study Treatment

The nutraceutical treatment consisted of 400 mg Taurisolo® twice daily. Taurisolo® is a nutraceutical supplement consisting of a polyphenol extract obtained from *Aglianico* cultivar grape, collected during the autumn 2018 harvest. Firstly, the Department of Pharmacy, University of Naples Federico II (Naples, Italy), provided the supplement formulation, then the large-scale production was accomplished by MB-Med Company (Turin, Italy). For the polyphenol extract production, grapes were extracted with water (50°C), and the solution was filtrated and concentrated and underwent a spray-drying process with maltodextrins as support (5–15%) to obtain a fine microencapsulated powder. The polyphenol profile of Taurisolo® was evaluated by High-Performance Liquid Chromatography-diodearray detector (HPLC-DAD, Jasco Inc., Easton, MD, USA) analysis using the method described by Giusti et al. (33): Ferulic acid 14.59 ± 0.98 μg/g, Resveratrol 12.55 ± 0.02 μg/g, Caffeic acid 35.00 ± 3.00 μg/g, p-coumaric acid 122.75 ± 2.77 μg/g, Rutin 98.81 ± 7.31 μg/g, Quercetin 135.41 ± 4.69 μg/g, Procyanidin B1 dimer 946.33 ± 55.20 μg/g, Procyanidin B2 dimer 645.89 ± 59.17 μg/g, Syringic acid 310.95 ± 0.01 μg/g, Epicatechin 1696.55 ± 109.60 μg/g, Gallic acid 199.46 ± 4.59 μg/g; Catechin 2499.04 ± 307.41 μg/g (34).

### TMAO Serum Levels Quantification

TMAO serum levels were quantified by the LC-MS method described by Annunziata et al. (35) and previously reported (13–15, 36). Briefly, to 80 μL of plasma were added 160 μL of methanol. The mixture was vortexed for 2 min, and then centrifuged at 12,000 g. The supernatant was collected and used for the LC-MS analysis. The LC-MS system used, and method conditions are detailed in Supplementary Materials.

### Serum Levels of Oxidative Stress-Related Biomarkers Quantification

Serum levels of reactive oxygen metabolites (D-ROMs) and oxidized low-density lipoproteins (oxLDL) as oxidative stress-related biomarkers were monitored. Both D-ROMs and oxLDL analyses were carried out on an automated analyzer (Free Carpe Diem, Diacron International, Grosseto, Italy) using relative commercial kits (Diacron International) according to the manufacturer's instructions, as previously reported (35, 37). Methods are detailed in Supplementary Materials.

### Statistics

All the experimental data were expressed as mean ± standard deviation (SD). Statistical analysis of data was carried out by the Student's *t*-test or Pearson correlation. The level of significance (α-value) was 95% in all cases (*P* < 0.05). The degree of linear relationship between two variables was measured using the Pearson product moment correlation coefficient (R). Correlation coefficients (R) were calculated using Microsoft Office Excel.

## Results

### Enrolment and Subject Attrition

**Figure 1:**
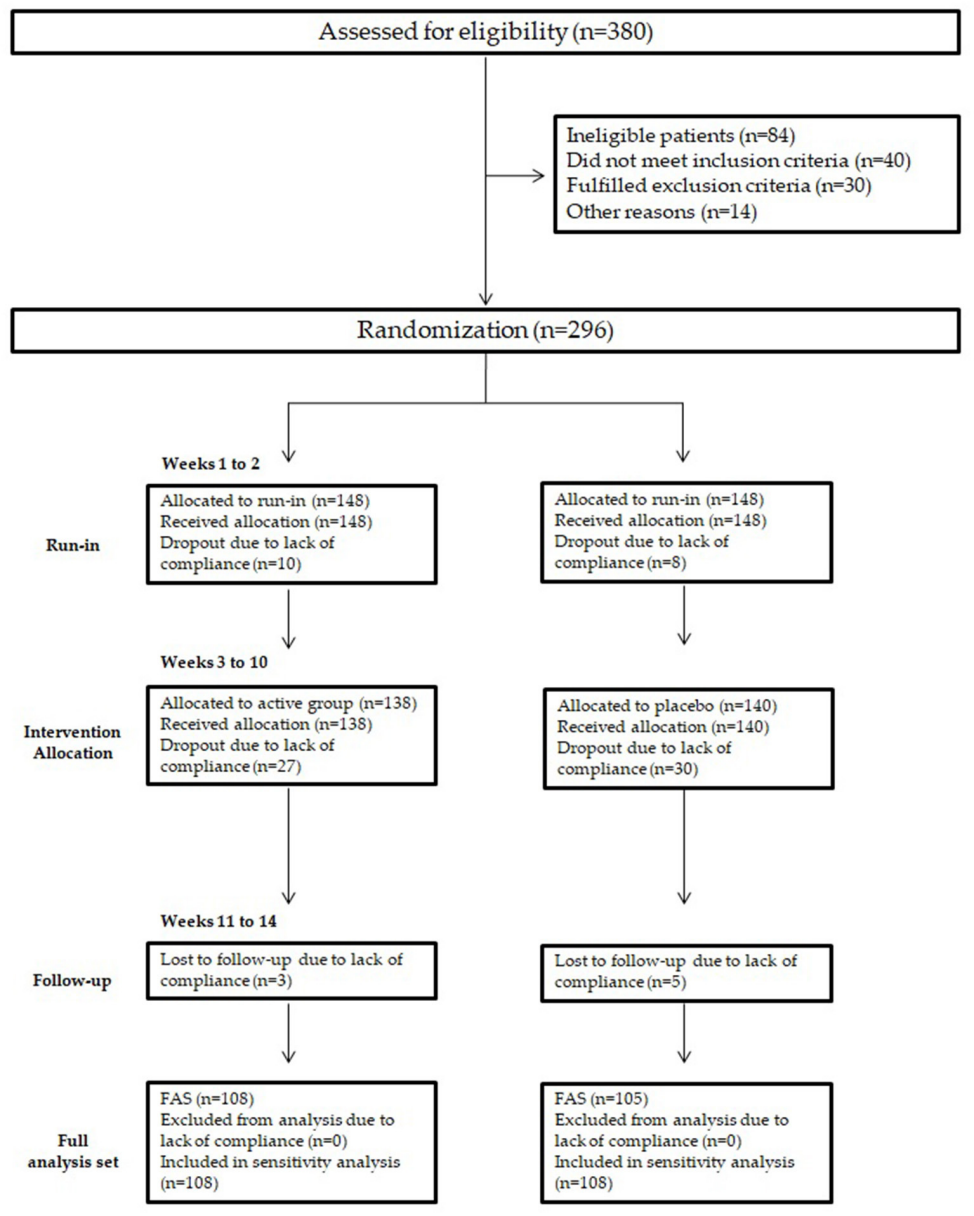
Study flowchart. Study flowchart, according to the consolidated standards of reporting trials (CONSORT). The diagram shows enrolment and primary efficacy endpoints based on patients' diaries, from prescreening to data collection; and the extent of exclusions, loss to follow-up, and completeness of. Diary documentation available across the entire trial period. FAS, Full analysis set.

Subjects were enrolled during the months January-March 2019. A total of 380 subjects were screened for eligibility; 84 subjects did not pass the screening stage. Overall, 296 subjects were randomized into active or placebo groups. Figure 1 shows the flow of participants through the trials together with the completeness of diary information over the entire treatment period. Figure 1 follows the CONSORT PRO reporting guideline (38).

### Baseline Characteristics of Study Participants

Table 1 reports the demographic and clinical characteristics assessed at the baseline visit of all study participants. Overall, 64% of subjects were male and, on average, middle-aged and overweight. Type 2 diabetes mellitus (T2DM) and hypertension were the most diagnosed diseases among the study participants (28 and 35%, respectively), and for this reason they were considered in further analysis.

**Table 1:** Baseline characteristics of study participants.

**Characteristic**	**All subjects (*n* = 213)**	**Active group (*n* = 108)**	**Placebo group (*n* = 105)**	***p*-value**
Age (year)	61.81 ± 11.98	60.94 ± 13.55	62.70 ± 10.11	0.287
Gender (M/F)	136/77	66/42	70/35	
BMI (kg/m^2^)	29.11 ± 4.18	28.79 ± 4.17	29.44 ± 4.19	0.256
Smokers [yes (%)]	53.52	51.54	55.00	χ^2^ = 0.245, *p* = 0.623
Physical activity [yes (%)]	31	32.47	29.50	χ^2^ = 0.207, *p* = 0.649
T2DM [yes (n.)]	60	28	32	χ^2^ = 0.545, *p* = 0.460
Hypertension [yes (n.)]	74	39	35	χ^2^ = 0.181, *p* = 0.670
TMAO (μM)	2.30 ± 2.05	2.19 ± 2.30	2.41 ± 1.75	0.440
oxLDL (μEq/L)	800.78 ± 185.61	795.96 ± 189.18	805.75 ± 185.75	0.701
D-ROMs (UCARR)	475.68 ± 129.15	477.08 ± 135.38	474.24 ± 123.04	0.873

### TMAO-Reducing Effect of Taurisolo®

**Figure 2:**
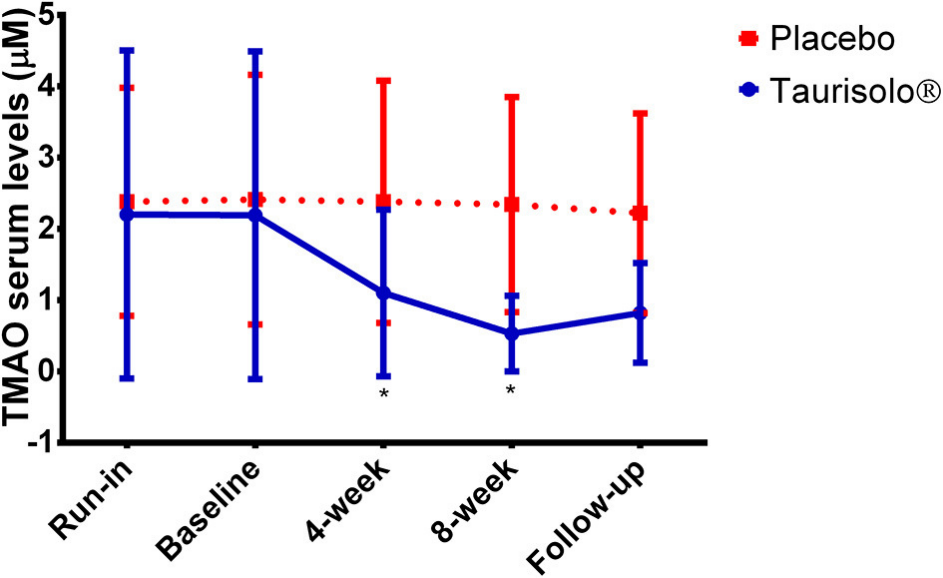
Graphical representation of the TMAO serum levels at the different time points. Values are expressed as mean ± standard deviation. Statistical significance was calculated with Student's *t*-test; *p* < 0.05 was considered as significant. *P < 0.0001, Taurisolo® group, compared to baseline.

TMAO serum levels were monitored in all study participants at run-in, before starting Taurisolo® treatment (baseline), after 4-week treatment with both Taurisolo® or placebo, after 8-week treatment with both Taurisolo® or placebo and after 4-week follow-up (Figure 2). TMAO serum levels significantly reduced after 4-week Taurisolo® treatment, from 2.91 ± 2.30 μM to 1.10 ± 1.17 μM (−49.78%, *p* < 0.0001), and after 8-week Taurisolo® treatment (0.53 ± 0.53 μM, −75.85%, *p* < 0.0001). No significant changes were observed in control group after treatment period with placebo.

Subjects in active group were stratified for (i) gender, (ii) diagnosis of diabetes or hypertension, (iii) body mass index (BMI), and (iv) age. The effects of Taurisolo® on TMAO serum levels are reported in Tables 2–5. Since no significant differences were observed in control group, we performed this stratification only for Taurisolo®-treated subjects.

**Table 2:** Serum levels of TMAO before and after 4-week and 8-week treatment with Taurisolo® in study participants stratified for gender.

	**Baseline**	**t-4wk**			**t-8wk**		
	**Mean ±SD**	**Mean ±SD**	**Δ% (vs. t0)**	***p* (vs. t0)**	**Mean ±SD**	**Δ% (vs. t0)**	***p* (vs. t0)**
Men (*n* = 66)	2.57 ± 2.71	1.43 ± 1.41	−44.23	<0.0001	0.50 ± 0.38	−80.59	<0.0001
Women (*n* = 42)	2.11 ± 1.53	1.21 ± 0.94	−42.89	<0.0001	0.61 ± 0.67	−71.22	<0.0001

*Statistical significance was calculated with Student's t-test; p < 0.05 was considered as significant*.

**Table 3:** Serum levels of TMAO before and after 4-week and 8-week treatment with Taurisolo® in study participants stratified for diagnosis of diabetes or hypertension.

	**Baseline**	**t-4wk**			**t-8wk**		
	**Mean ±SD**	**Mean ±SD**	**Δ% (vs. t0)**	***p* (vs. t0)**	**Mean ±SD**	**Δ% (vs. t0)**	***p* (vs. t0)**
Diabetes (*n* = 28)	3.71 ± 2.81	2.11 ± 1.15	−43.15	<0.001	0.73 ± 0.67	−80.31	<0.0001
Hypertension (*n* = 39)	3.02 ± 2.87	1.79 ± 1.42	−40.84	0.0001	0.64 ± 0.64	−78.88	<0.0001
No-diabetes/No-hypertension (*n* = 41)	1.32 ± 1.10	0.66 ± 0.63	−49.82	<0.0001	0.40 ± 0.32	−69.92	<0.0001

*Statistical significance was calculated with Student's t-test; p < 0.05 was considered as significant*.

**Table 4:** Serum levels of TMAO before and after 4-week and 8-week treatment with Taurisolo® in study participants stratified for BMI.

	**Baseline**	**t-4wk**			**t-8wk**		
	**Mean ±SD**	**Mean ±SD**	**Δ% (vs. t0)**	***p* (vs. t0)**	**Mean ±SD**	**Δ% (vs. t0)**	***p* (vs. t0)**
Normal weight (*n* = 57)	0.96 ± 0.61	0.48 ± 0.29	−50.27	<0.0001	0.33 ± 0.23	−65.53	<0.0001
Overweight (*n* = 21)	1.84 ± 0.59	1.30 ± 0.47	−29.27	<0.001	0.54 ± 0.29	−70.75	<0.0001
Grade I obesity (*n* = 16)	3.62 ± 0.88	2.04 ± 0.76	−43.68	<0.0001	0.72 ± 0.40	−80.12	<0.0001
Grade II obesity (*n* = 14)	7.06 ± 2.45	3.75 ± 0.73	−46.84	0.001	1.14 ± 1.00	−83.86	<0.0001

*Statistical significance was calculated with Student's t-test; p < 0.05 was considered as significant*.

**Table 5:** Serum levels of TMAO before and after 4-week and 8-week treatment with Taurisolo® in study participants stratified for age.

	**Baseline**	**t-4wk**			**t-8wk**		
	**Mean ±SD**	**Mean ±SD**	**Δ% (vs. t0)**	***p* (vs. t0)**	**Mean ±SD**	**Δ% (vs. t0)**	***p* (vs. t0)**
18–40 years (*n* = 14)	1.89 ± 0.32	0.78 ± 0.56	−58.92	<0.0001	0.75 ± 0.33	−60.08	<0.0001
41–60 years (*n* = 31)	2.53 ± 3.16	1.29 ± 1.32	−49.13	<0.001	0.65 ± 0.80	−74.24	<0.001
>61 years (*n* = 63)	2.55 ± 1.95	1.41 ± 1.29	−44.73	<0.0001	0.46 ± 0.34	−81.87	<0.0001

*Statistical significance was calculated with Student's t-test; p < 0.05 was considered as significant*.

### The Effect of Taurisolo® in Reducing Oxidative Stress-Related Biomarkers

**Figure 3:**
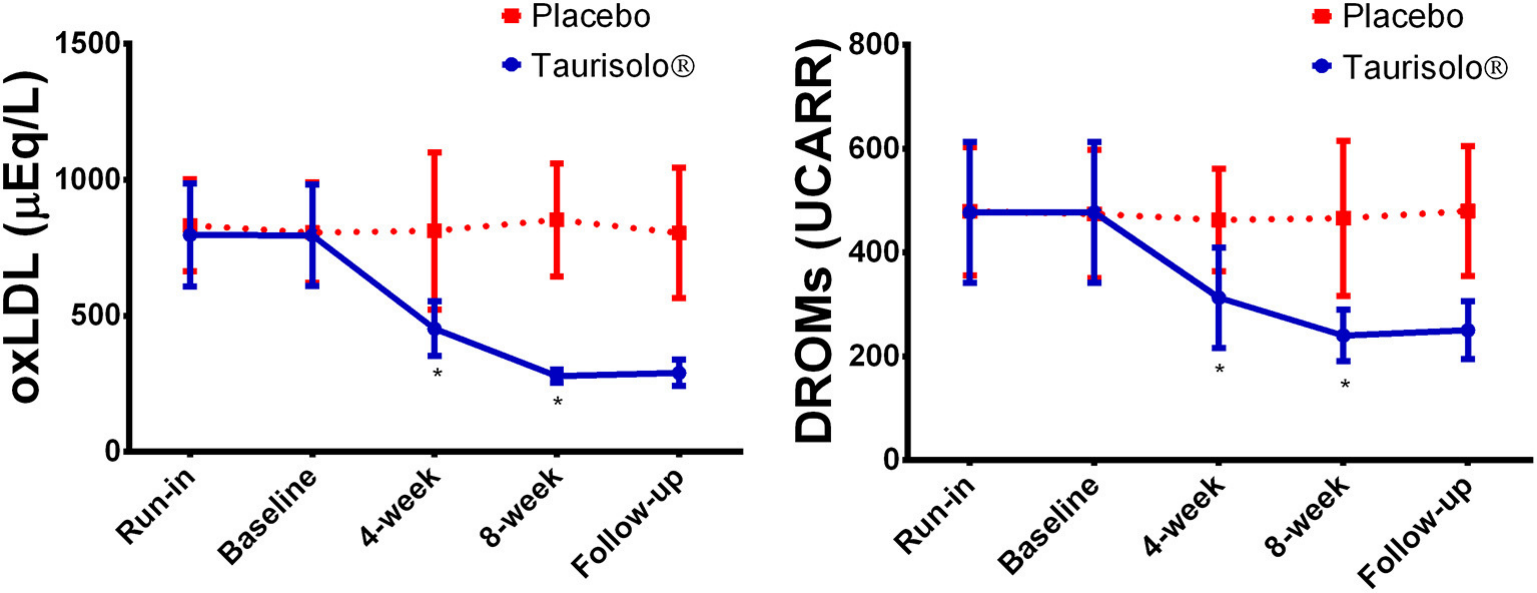
Graphical representation of the oxidative stress-related biomarkers serum levels at the different time points. Values are expressed as mean ± standard deviation. Statistical significance was calculated with Student's *t*-test; *p* < 0.05 was considered as significant. *P < 0.0001, Taurisolo® group, compared to baseline.

Serum levels of oxLDL and D-ROMs were monitored in all study participants at run-in, before starting intervention period (baseline), after 4-week treatment with Taurisolo® or placebo, after 8-week treatment with Taurisolo® or placebo and after 4-week follow-up (Figure 3). oxLDL levels significantly reduced after 4-week Taurisolo® treatment, from 795.95 ± 186.74 μEq/L to 452.78 ± 100.72 μEq/L (−43.12%, *p* < 0.0001), and after 8-week Taurisolo® treatment (278.15 ± 24.48 μM, −65.05%, *p* < 0.0001). Similar trend was also observed for D-ROMs levels that significantly reduced after 4-week Taurisolo® treatment, from 477.08 ± 135.38 UCARR to 313.09 ± 96.70 UCARR (−34.37%, *p* < 0.0001), and after 8-week Taurisolo® treatment (240.07 ± 49.44 UCARR, −49.68%, *p* < 0.0001). No significant changes were observed in control group after treatment period with placebo.

### Correlation Analysis Between Variations of TMAO, oxLDL and DROMs After 8-Week Treatment With Taurisolo®

A Pearson's correlation analysis was performed between (i) serum levels of TMAO, oxLDL and D-ROMs (at baseline, after 4-week and 8-week Taurisolo® treatment) (Table 6) and (ii) variations of these three parameters during both the 4-week and 8-week treatment periods (indicated as Δ% (baseline-t-4wk) and Δ% (baseline-t-8wk, respectively) (Table 7). Since no significant differences were observed in control group, we performed this correlation analysis only for Taurisolo®-treated subjects.

**Table 6:** Correlation analysis between TMAO, oxLDL, and D-ROMs serum levels in all study participants.

	**TMAO baseline**		**TMAO t-4wk**		**TMAO t-8wk**	
	**Coefficient**	***p-*value**	**Coefficient**	***p-*value**	**Coefficient**	***p-*value**
**oxLDL**						
Baseline	0.975	<0.0001	–	–	–	–
t-4wk	–	–	0.969	<0.0001	–	–
t-8wk	–	–	–	–	0.972	<0.0001
**D-ROMs**						
Baseline	0.975	<0.0001	–	–	–	–
t-4wk	–	–	0.946	<0.0001	–	–
t-8wk	–	–	–	–	0.907	<0.0001

**Table 7:** Correlation analysis between variations of TMAO, oxLDL, and D-ROMs serum levels in all study participants.

	**TMAO** **Δ% (Baseline-t-4wk)**		**TMAO** **Δ% (Baseline-t-8wk)**	
	**Coefficient**	***p-*value**	**Coefficient**	***p-*value**
**oxLDL**				
Δ% (Baseline-t-4wk)	0.757	<0.0001	–	–
Δ% (Baseline-t-8wk)	–	–	0.221	<0.05
**D-ROMs**				
Δ% (Baseline-t-4wk)	0.654	<0.0001	–	–
Δ% (Baseline-t-8wk)	–	–	0.178	0.066

## Discussion

**Figure 4:**
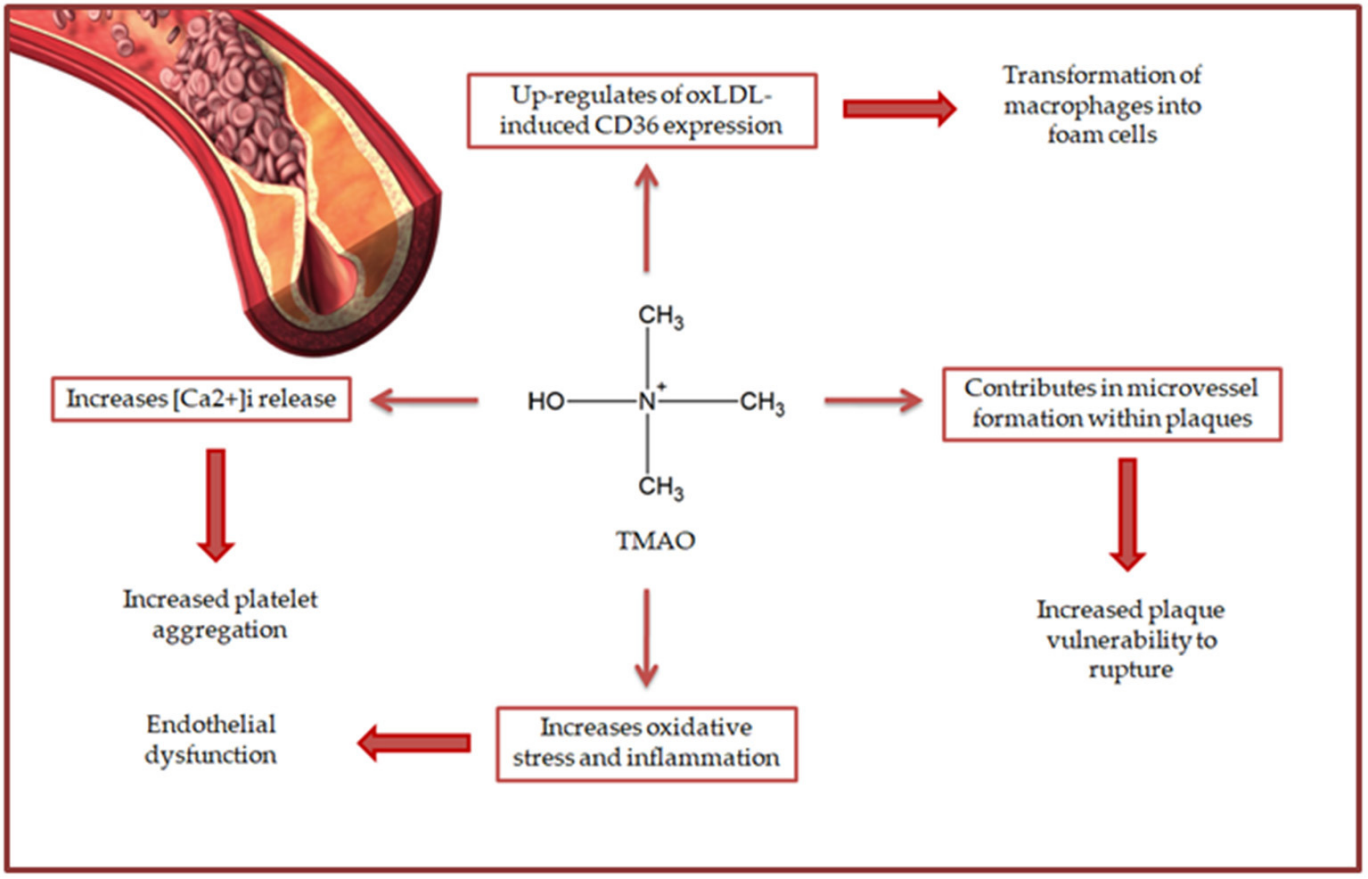
The role of TMAO in atherosclerosis development and progression.

TMAO is a novel proatherogenic factor playing a crucial role in development and progression of atherosclerosis *via* different mechanisms (2), as schematically reported in Figure 4.

TMAO has been reported to up-regulate the expression of scavenger receptor CD36 induced by oxLDL in macrophages (39, 40); this promotes the transformation of macrophages into foam cells (3, 8). This mechanism suggests the participation of TMAO in the early stages of atherosclerotic plaque formation. Once formed, atherosclerotic plaques are particularly vulnerable to rupture (41), resulting in increased risk of cardiovascular events (42, 43). A cohort study on patients with coronary artery disease reported higher TMAO plasma levels in patients with plaque rupture than in non-plaque rupture ones (44), suggesting a close involvement of TMAO in plaque instability (2). More specifically, TMAO seems to contribute to microvessel formation within the plaques, increasing their vulnerability to rupture (45). Also, TMAO levels have been found to correlate positively with the percentage of intermediate CD14++CD16+ monocytes (46). This subset promotes both (i) new vessel formation within the plaque *via* a pro-inflammatory mechanism (47) and (ii) plaque rupture *via* overproduction of matrix metalloproteinases (48). Notably, TMAO also exerts the pro-thrombrotic effect *via* triggering the release of [Ca^2+^]_i_, resulting in increased platelet aggregation (49).

Recent evidence also indicated TMAO as key actor playing a role in endothelial dysfunction (2). In particular, TMAO seems to be involved in mechanisms increasing (i) OxS, *via* promoting ROS generation and nitrogen oxide reduction (50, 51) and (ii) inflammation, *via* down-regulation of Il-10 (50). Both pro-oxidant and pro-inflammatory activities of TMAO have been indicated as responsible for the reduction in circulating endothelial progenitor cell observed in stable angina patients (52).

Overall, this evidence clarifies the role played by TMAO in atherosclerotic risk and suggests this compound as a potential target for management of atherosclerosis.

### Taurisolo® Reduces Serum Levels of TMAO

The first aim of our study was to prove that chronic administration of Taurisolo® reduced TMAO serum levels in a large number of subjects. Results herein presented are in line with our previous observations of the TMAO-reducing effect of Taurisolo®. Both in healthy (36) and overweight/obese subjects (35), indeed, we reported the ability of our nutraceutical formulation to reduce the serum levels of TMAO after 4- and 8-week treatment, respectively. In this study, Taurisolo® reduced TMAO levels by 49.78% after 4 weeks, and 75.85% after 8 weeks in all study participants. Similar trend was also maintained when subjects were stratified for (i) gender, (ii) diagnosis of diabetes or hypertension, (iii) body mass index (BMI), and (iv) age, as reported in Tables 2–5.

Besides the very large number of papers published on TMAO in last decades, no mechanisms have been proposed for the potential TMAO-reducing effect of drugs or natural compounds. However, studies reported in literature allow speculating possible mechanisms of action. In particular, we hypothesized two different ways by which polyphenols may contribute to reduce TMAO serum levels: antioxidant and/or microbiota remodeling activities.

Polyphenols exert their antioxidant activity through direct and indirect mechanisms (30), in particular transferring an electron to free radicals, acting, thus, as “electron donors” (31, 32). In contrast, TMAO has been reported to act as an “electron acceptor” in bacteria (53), leading to a so-called “redox hypothesis,” where both polyphenols and TMAO are two actors of the same redox reaction occurring at blood levels, and resulting in a chemical reduction of TMAO to TMA.

The antioxidant potential of Taurisolo® has been previously reported both in human (35) and rats (54). Similarly, our previous evidence demonstrated a high bioavailability of Taurisolo^®^ polyphenols (36), due to the use of microencapsulation in maltodextrins, a useful strategy to enhance the absorption of polyphenols across the intestinal barriers (26). It appears clear, thus, that, whether confirmed, the “redox hypothesis” might be a possible mechanism of action for the TMAO-reducing effect of Taurisolo^®^.

In addition to this hypothesis, we also proposed the so-called “microbiota hypothesis” based on the ability of polyphenols to modulate the growth of different bacterial strains. In particular, it is reported in literature that grape polyphenols are capable to reduce the growth of TMA-producing bacteria (i.e., *Clostridia* and *Bacteroides*) (55, 56) and to increase that of non-TMA-producing ones (i.e., *Lactobacillus* and *Bifidobacterium*) (29), resulting in a remodeling of the gut microbiota. According to our “microbiota hypothesis,” after chronic oral administration, Taurisolo® polyphenols might contribute to reduction of TMAO serum levels *via* blocking the colonic production of its precursor, TMA. This, in turn, might be operated by the antimicrobial activity against TMA-producing strains or a potential inhibition of their metabolism, for example down-regulation/inhibition of target genes/enzymes, such as TMA lyase, carnitine oxygenase and betaine reductase, responsible for the TMA release from food components. This hypothesis is again corroborated by the nature of our nutraceutical formulation, administered in an acid-resistant form. According to our previous investigations, the use of acid-resistant formulation protects polyphenols against degradation during the transit in the gastrointestinal tract, and allows polyphenols reaching the intestine in an active form (57).

Undoubtedly, both redox and microbiological hypothesis, need to be further investigated.

### Taurisolo® Reduces Serum Levels of Oxidative Stress-Related Biomarkers

In addition to the TMAO-reducing effect, we also investigated the ability of Taurisolo® to reduce oxLDL and D-ROMs as OxS-related biomarkers, since OxS is historically recognized as one of the main risk factors for CVD. In this study, both oxLDL and D-ROMs levels significantly reduced by 43.12 and 34.37% after 4-week and by 65.05 and 49.68% after 8-week treatment with Taurisolo®, respectively.

Besides the well-established antioxidant potential of polyphenols, several studies reported the ability of polyphenols to contrast atherosclerosis progression *via* reduction of LDL oxidizability (58, 59). In particular, polyphenols act as hydrogen donors to α-tocopherol radicals, resulting in prevention of LDL oxidation (60). Also, mechanistic studies reported that red wine polyphenols inhibited copper-catalyzed LDL oxidation (61). In line with both this evidence and data herein reported, we previously demonstrated the ability of chronic treatment with Taurisolo® to reduce circulating oxLDL in overweight/obese subjects (35). As in the present study, circulating oxLDL were monitored using the LP-CHOLOX test (Diacron, Grosseto, Italy), aimed to measure the levels of lipid peroxidation-derived hydroperoxides, mainly represented by oxidized cholesterol. According to the manufacturer's instructions, oxLDL levels are defined: normal (with values ≤ 599 μEq/L), slightly high (with values ranging from 600 to 799 μEq/L), moderately high (with values ranging from 800 to 999 μEq/L) and very high (with values ≥1,000 μEq/L) (62, 63). In agreement with this classification, our results showed that a 8-week treatment with Taurisolo® significantly reduced serum levels of oxLDL in all study participants from a slightly high to normal level.

DROMs are stable and quantifiable oxygen metabolites produced from free radical attacks operated at the expense of biomolecules. The test used in the present study is based on the concept that, according to the Fenton's reaction, DROMs contained in a serum generate, in presence of iron, alkoxyl (R–O^*^) and peroxyl (R–OO^*^) radicals which, in turn, oxidize an alkyl-substituted aromatic amine, producing a pink-colored derivative ([A–NH2^*^]+), photometrically quantified (64–67). DROMs, thus, are useful biomarkers of OxS, determined on the basis of the following ranges: (i) normal: 250–300 UCARR, (ii) border-line: 300–320 UCARR, (iii) low level of oxidative stress: 321–340 UCARR, (iv) middle level of oxidative stress: 341–400 UCARR, (v) high level of oxidative stress: 401–500 UCARR and (vi) very high level of oxidative stress: >500 UCARR, where 1 UCARR = 0.08 mg H_2_O_2_/dL (64– 67). According to this classification, our results showed that chronic treatment with Taurisolo® significantly reduced OxS in all study participants, from a very high to normal level.

Interestingly, our correlation analysis demonstrated that TMAO serum levels correlated positively with circulating oxLDL and DROMs at baseline and after 4- and 8-week treatment with Taurisolo® (Table 6); similarly, variations of TMAO serum levels during the nutraceutical treatment also correlated positively with variation of oxLDL and DROMs levels (Table 7). This suggests, on one hand, the link between this microbiota metabolite and OxS-related biomarkers, and on the other hand that chronic treatment with Taurisolo® may represent a valid nutraceutical approach for cardioprotection.

This study, however, is not without limitations. Firstly, specific cardiovascular examination aimed to test both progression or regression of atherosclerotic changes have not been performed; this is mainly due to both aims and design of this study. Also, in this study we did not consider preoperative subjects since no clinical evaluations of the atherosclerotic risk were performed. More specifically, this is pilot study aimed to demonstrate the efficacy of Taurisolo® to reduce specific serum biomarkers that have been profoundly associated with the atherosclerotic risk. However, further clinical trials investigating the effects of Taurisolo® on plaque reduction and atherosclerosis progression in high-risk patients are needed, and currently ongoing. Moreover, the study of the microbiota would be necessary to confirm our proposed microbiological hypothesis. In this sense, however, according to our results it is plausible to speculate that the observed reduction in TMAO serum levels might be due to diminished intestinal production of TMA that, in turn, results from a Taurisolo® polyphenols-induced microbiota remodeling. On the other hand, the relatively high sample size, the presence of a control group and the consistent results and correlation analysis may be useful for physicians, informing them about a novel nutraceutical remedy for the treatment of high cardiovascular risk-subjects.

## Data Availability Statement

The original contributions presented in the study are included in the article/Supplementary Material, further inquiries can be directed to the corresponding author/s.

## Ethics Statement

The studies involving human participants were reviewed and approved by Scientific Ethics Committee of AO Rummo Hospital (Benevento, Italy). The patients/participants provided their written informed consent to participate in this study.

## Author Contributions

MD'A, DC, GT, and EN: conceptualization, validation, and visualization. GA and RC: data curation. GA, RC, and MM: formal analysis. EN: funding acquisition and supervision. GA, RC, MM, MD'A, DC, GT, and EN: investigation. GA: writing—review and editing. All authors contributed to the article and approved the submitted version.

## Conflict of Interest

The authors declare that the research was conducted in the absence of any commercial or financial relationships that could be construed as a potential conflict of interest.
