# Supplementary material for: Taurisolo®, a Grape Pomace Polyphenol Nutraceutical Reducing the Levels of Serum Biomarkers Associated With Atherosclerosis
Source: Front Cardiovasc Med. 2021 Jul 19;8:697272. doi: 10.3389/fcvm.2021.697272 (PMC8326362; doi:10.3389/fcvm.2021.697272)
Supplement: Supplementary file 1 [file Data_Sheet_1.doc]

Supplementary Material

***Materials and methods***

*TMAO quantification*

The HPLC system Jasco Extrema LC-4000 system (Jasco Inc., Ithaca, NY) was coupled to an Advion Expression mass spectrometer (Advion Inc., Ithaca, NY) equipped with an ESI source, operating in positive ion mode. The separation of the analytes was performed using a Luna Hilic (5µ particle size, 150x3mm) and security guard colon both supplied by Phenomenex (Torrance, CA, USA) were used. The column temperature was maintained at 60 °C during analysis. Mobile phase A composition: 0.15% formic acid in water containing a final concentration of 10 mM ammonium acetate; mobile phase B composition: methanol. Mobile phases ratio was 80:20 (A:B), run isocratically at a flow rate of 0.35 mL/min for 6 min, with a 5 µl injection volume.

*D-ROMs test (Free Carpe Diem, Diacron International, Grosseto, Italy)*

10µl of serum were transferred into 1cm cuvettes containing 1ml of R_2_ reagent (acetate buffer, pH4.8). The sample-containing mixture was gently mixed and 20µl of R_1_ reagent (a chromogenic mixture consisting of aromatic alkyl-amine, A-NH_2_) were added. Cuvettes were mixed by inversion and samples were read at 546nm (5 min, 37°C) on an automated analyzer.

*LP-CHOLOX test (Free Carpe Diem, Diacron International, Grosseto, Italy)*

10µl of serum were added in a plastic tube containing 1ml of R_1_ reagent (indicators mixture) and two drops of R_2_ reagent (reduced iron) were transferred. The mixture was mixed by shaking, incubated at 37°C for 2 min and centrifuged at 1,400 g for 2 min. Supernatants were transferred into 1cm cuvettes and read at 505nm (37°C) on an automated analyzer. Blank was prepared following the same procedure, without the addition of sample.

*Study population and protocol*

Study participants were recruited by the Cardarelli Hospital (Naples, Italy). Subjects were enrolled in January-April 2019. All subjects underwent a standardised physical examination, assessment of medical history (for up to five years before enrolment), laboratory examination, measurement of blood pressure and heart rate, and evaluation of BMI. Body mass index (BMI) was calculated from body height and body weight. Body fat percentage was measured using a body composition analyzer (TBF-310, Tanita Corp., Tokyo, Japan) and systolic blood pressure, diastolic blood pressure, and heart rate, were measured using a HBP-9020 (OMRON COLIN Corp., Tokyo, Japan). At each clinic visit, subjects had to complete three self-administered questionnaires on quality of life aspects, and their diaries were checked for data completeness and quality of documentation to ensure subject comprehension of the diary items. Subjects aged 18–75 years with body mass index (BMI) ≥ 18.5 kg/m^2^ were eligible for enrolment.

The subjects received oral and written information concerning the study before they gave their written consent. Protocol, letter of intent of volunteers, and synoptic document about the study were submitted to the Scientific Ethics Committee of AO Rummo Hospital (Benevento, Italy). The study was approved by the committee (protocol 106 n. 123512 of 18/06/2018), and carried out in accordance with the Helsinki declaration of 1964 (as revised in 2000). This study is listed on the ISRCTN registry (www.isrctn.com) with ID ISRCTN10794277 (doi: 10.1186/ISRCTN10794277). The subjects were asked to make records in an intake-checking table for the intervention study and side effects in daily reports.

Subjects were informed not to drink alcohol or perform hard physical activity 48 h prior to blood sampling. Participants maintained their usual dietary and lifestyle patterns throughout the study.

*Study procedures*

Participants arrived at the research centre in the morning after 12 h of fasting. All blood samples were taken in the morning and immediately after measurement of heart rate and blood pressure. Blood samples were collected from each participant before administration of the reference glucose solutions and the treatment beverages, in 3-mL EDTA-coated tubes (Becton–Dickinson, Plymouth, UK). Plasma was immediately isolated by centrifugation (20 min, 2.200 g, 4 °C). All biochemical analyses including fasting plasma glucose, total cholesterol, fasting plasma TG were performed with a Roche Modular Analytics System in the Central Biochemistry Laboratory of our Institution. Low-Density Lipoprotein (LDL) cholesterol and HDL cholesterol were determined by a direct method (homogeneous enzymatic assay for the direct quantitative determination of LDL and HDL cholesterol).

*Statistics*

*Methodology*

Unless otherwise stated, all of the experimental results were expressed as mean ± SEM. Statistical analysis of data was performed by the Student’s t test or Pearson correlation. The statistic heterogeneity was assessed by using Cochran’s test (p<0.1). The I2 statistic was also calculated, and I2>50% was considered as significant heterogeneity across studies. A random-effects model was used if significant heterogeneity was shown among the trials. Otherwise, results were obtained from a fixed-effects model. SD values were calculated from standard errors, 95% CIs, p-values, or t if they were not available directly. Previously defined subgroup analyses were performed to examine the possible sources of heterogeneity within these studies and included health status, study design, type of intervention, duration, total nutraceutical dose, and Jadad score. Treatment effects were analysed using PROC MIXED with treatment and period as fixed factors, subject as random factor and baseline measurements as covariates, and defined as weighted mean difference and 95% CIs calculated for net changes in fecal and serum parameters, and blood pressure values. Data that could not meet the criteria of variance homogeneity (Levenes test) and normal distribution (determined by residual plot examination and Shapiro–Wilks test) even after log transformation were analysed by a nonparametric test (Friedman). The level of significance (α-value) was 95% in all cases (P < 0.05).

*Analysis set*

The full analysis set population included all randomised subjects, and subjects who did not fail to satisfy a major entry criterion. The per protocol set consisted of all subjects who did not substantially deviate from the protocol. This group included subjects for whom no major protocol violations were detected (for example, poor compliance, errors in treatment assignment).

*Statistics*

All of the experimental data were expressed as mean ± SEM. Statistical analysis of data was carried out by the Student’s t test or Pearson correlation. The level of significance (α-value) was 95% in all cases (P < 0.05). The degree of linear relationship between two variables was measured using the Pearson product moment correlation coefficient (R). Correlation coefficients (R) were calculated using Microsoft Office Excel.
